# Efficacy of an Internet-based intervention for job stress and burnout among medical professionals: study protocol for a randomized controlled trial

**DOI:** 10.1186/s13063-019-3401-9

**Published:** 2019-06-10

**Authors:** Ewelina Smoktunowicz, Magdalena Lesnierowska, Roman Cieslak, Per Carlbring, Gerhard Andersson

**Affiliations:** 1 Department of Psychology, SWPS University of Social Sciences and Humanities, Chodakowska 19/31, 03–815 Warsaw, Poland; 2 Trauma, Health, and Hazards Center, University of Colorado, Colorado Springs, CO USA; 3 Department of Psychology, Stockholm University, Stockholm, Sweden; 4 Department of Psychology, University of Southern Denmark, Odense, Denmark; 5 Department of Behavioural Sciences and Learning, Linköping University, Linköping, Sweden; 6 Department of Clinical Neuroscience, Karolinska Institutet, Stockholm, Sweden

**Keywords:** Job stress, Job burnout, Internet-based intervention, Self-efficacy, Perceived social support, Resources, e-mental health, Randomized controlled trial, Health professionals, Occupational health

## Abstract

**Background:**

Medical professionals are at high risk of job stress and burnout. Research shows that work-related stress can be reduced through enhancing psychological resources, in particular, self-efficacy and perceived social support. These psychological resources can operate either individually or sequentially: in line with the cultivation hypothesis, self-efficacy precedes and cultivates perceived social support, whereas according to the enabling hypothesis it is perceived social support that comes first and enables self-efficacy. Based on this theoretical framework we developed an internet-based intervention, Med-Stress, dedicated to healthcare providers and aimed at reducing job stress and burnout. Med-Stress contains two modules that enhance self-efficacy and perceived social support, which are tested in four variants reflected in four study conditions. We expect that sequential enhancement of resources: self-efficacy and social support or social support and self-efficacy will yield larger posttest results than individual enhancement.

**Methods:**

In this four-arm randomized controlled trial we will test four variants of the Med-Stress intervention. The trial is open for professionally active medical providers aged at least 18 years (*N* = 1200) with access to an Internet-connected device. We will compare the effects of two experimental conditions reflecting cultivation and enabling effects of self-efficacy and perceived social support (sequential enhancement of resources), and two active controls strengthening self-efficacy or perceived social support. Job stress and job burnout will be the primary outcomes, whereas depression, job-related traumatic stress, and work engagement will be secondary ones. Additionally, we will measure perceived social support, self-efficacy to manage job stress and burnout, and the ability to obtain social support, exposure to traumatic events, and users’ expectancy and credibility of the intervention. All assessments will be applied before the intervention, at posttest (at 3 or 6 weeks depending on the study condition), and at 6-month and 12-month follow up. In the case of experimental groups, additional measurements will be taken after enhancing each resource.

**Discussion:**

Resource-based interventions are relatively context-free and could potentially benefit medical professionals across the field. If Med-Stress is successful, its most effective variant could be implemented in the healthcare system as a standalone, supportive tool for employees.

**Trial registration:**

ClinicalTrials.gov, NCT03475290 Registered on 23 March 2018.

**Electronic supplementary material:**

The online version of this article (10.1186/s13063-019-3401-9) contains supplementary material, which is available to authorized users.

## Background

A demanding work environment can detrimentally affect employees’ mental health, leading to job stress and burnout [1]. Job stress is a response to employees’ perceptions that their work demands exceed their ability to cope [2]. When this becomes a chronic state or is not adequately coped with, job stress can adversely affect employees, organizations and societies [3]. Concurrently, over the past four decades, the focus of occupational psychology has expanded to include job burnout as an indicator of mental health at work. Originally defined as a syndrome comprising emotional exhaustion, depersonalization, and decreased personal accomplishment [4], nowadays the popular view is that only the first two dimensions are required to recognize and measure job burnout [5]. Burned-out employees suffer from numerous physical and psychological conditions [6], commit more errors at work [7], and can “infect” others with their burnout [8]. Although job stress and burnout are rooted in the work environment, they can spill over into other life domains [9]. Estimating the prevalence of job stress and burnout is difficult and few countries recognize job burnout as a legitimate medical diagnosis (with some exceptions such as the Swedish version of International Classification of Diseases (ICD)-10 [10]). Thus, the distinction between job burnout and conditions with similar symptoms like fatigue, depression, and anxiety is often difficult to make [11], and can lead to classifying job burnout as another condition. Regardless of the specific diagnosis, the burden of job-related mental health problems among all professions involves direct costs of treatment but also indirect ones resulting from sick leave absences, job turnover, and early retirement, among others [12], making it a matter of public interest.

Although today we know that job stress and burnout are not limited to the helping professions, healthcare providers are still considered a vulnerable group [13]. This is particularly significant from the public standpoint because most of us become patients at some point in our lives. We surveyed 744 health workers in a pre-implementation study and found that 70% of the responders had experienced stress-related emotional and health problems themselves or observed them in their colleagues, while 60% declared that the symptoms of job burnout had become ubiquitous in their work environments. Moreover, 90% of the responders lacked institutional support in preventing stress and burnout (Lesnierowska M, Smoktunowicz E, Puchalska M, Cieslak R: Internet-based intervention for occupational stress among medical professionals: preimplementation study, unpublished report). These findings are in line with a recent systematic review on job burnout among physicians, which reported a prevalence of up to 80.5% [14], and with the 6th European Working Conditions Survey, which found that the health sector scored the highest on the so-called work intensity index comprising five types of job demands [1]. Studies conducted in various parts of the world [15, 16] confirm that stress and burnout are vital problems for medical professionals across the world, regardless of economic, system, and cultural differences. There is thus a pressing need for effective and widespread workplace interventions that meet the needs of this occupational group.

Over the past 25 years, there has been a rise in Internet-based mental health interventions that are delivered via new technologies (e.g., Internet, mobile apps, virtual reality). Occupational Internet-based interventions follow those dedicated to treating clinical conditions such as depression, anxiety, and phobias [17, 18]. They usually adopt the cognitive-behavioral therapy (CBT) framework and select general mental health conditions as outcomes; usually stress, depression, and anxiety [19]. We argue, however, that workplace interventions need to be derived from theoretical models developed to explain and predict work-specific conditions. For instance, interventions dedicated to targeting contextual factors at work could employ an influential job demands-resources model [5], whereas those focused on individual characteristics might adopt conservation of resources theory [20, 21]. Additionally, the outcomes should be representative of the work environment, such as job burnout or work engagement, rather than depression and anxiety, which tend to span all life domains. These issues have largely remained unaddressed, although there are notable exceptions (e.g., [22]). Evidence suggests that occupational Internet-based interventions do work. A few trials have succeeded in reducing job burnout [22–25], and results of meta-analyses demonstrate that they are successful in enhancing psychological well-being and increasing work effectiveness [26], and in reducing mental health problems such as stress and depression [19]. Work engagement, a positive counterpart of job burnout, reflecting vigor, absorption, and dedication to work [27], has been targeted less often [22, 25]. All the above listed outcomes are applicable in the occupational context, regardless of the demands of a given profession. However, healthcare providers experience unique demands such as indirect traumatic exposure (e.g., caring for trauma survivors) that can result in job-related traumatic stress. Although numerous Internet-based interventions address consequences of traumatic exposure in populations such as veterans (e.g., [28]), few of these focus on employees [24, 29].

Workplace interventions usually take into account the specific environmental factors, which maximizes the fit with their receivers’ needs but makes them difficult or impossible to generalize. An alternative is intervention targeting individual and social resources. The obvious downside of such interventions is that they do not match the needs exactly, but on the upside, have a greater chance of being relevant to many employees across a variety of workplaces. In other words, resource-based interventions can be beneficial for more employees, but might not address all their issues. According to conservation of resources theory [20], resources are “those objects, personal characteristics, conditions, or energies that are valued in their own rights, or that are valued because they act as conduits to the achievement or protection of valued resources” (p. 339). In this study we will consider two psychological resources, self-efficacy and perceived social support, the conduits to prevent and manage job stress and burnout, as well as expect their role in increasing work engagement, and decreasing depression and job-related traumatic stress.

Self-efficacy, a crucial concept in social cognitive theory [30], determines how people feel, think, and behave, and reflects their belief in being able to perform competently and achieve set goals despite obstacles. It allows the prediction of how employees will cope with work stress [31] and was found to have a medium, negative relationship with job burnout [32]. Apart from general self-efficacy, we distinguish context- or domain-specific self-efficacy, which is considered better at explaining and predicting outcomes [33]. As the primary outcomes in this study are job stress and burnout, we are interested in self-efficacy to cope with those. Additionally, as we explain later in this paper, we focus on the relationship between self-efficacy and perceived social support, and thus introduce self-efficacy to obtain social support.

Due to numerous definitions of social support, it now operates as an umbrella term, and authors of each study are expected to be precise in presenting their understanding of the concept. Our focus is on perceived social support, which we define as a reflection of the belief that help will be available when needed [34]. Compared with the support that is actually received, it is less dependent on environmental factors and more stable over time [35], which makes it a uniquely suitable part of the intervention that is based on enhancing individual resources, regardless of specific workplace context. Social support was found to play a number of roles in the process of job stress, from changing the perception of demands to moderating the strain-stress relationship [36], and a small negative relationship was found between perceived social support and job burnout [21].

Enhancement of perceived social support and self-efficacy was the focus of a previous intervention, Helpers’ Stress [25, 29], which was dedicated to professionals who worked with trauma survivors, and was found to be successful in reducing their job burnout and increasing work engagement. However, less is known about the joint impact of self-efficacy and perceived social support on mental health indicators. According to one of the predictions, the cultivation hypothesis, self-efficacy is a resource that comes first as it allows for reaching out to other people to obtain support when needed [37]. On the other hand, the enabling hypothesis proposes that that it is the perception of support availability or directly receiving help that enhances the sense of self-efficacy [38]. Both arguments have found considerable empirical support, however, mainly in the context of health behavior change [39], stress recovery [40], and work engagement [41]. What is lacking is experimental evidence from research conducted in an occupational setting and we aim to address this gap.

To summarize, the goal of this study is to test the efficacy of an internet-based intervention, Med-Stress, targeted mainly at reducing job stress and burnout among medical professionals, through the individual and sequential enhancement of self-efficacy and perceived social support. Med-Stress comprises two main modules - one dedicated to self-efficacy, and the other to perceived social support - that are then tested in four configurations that translate into four study conditions. Both modules are included in two of these conditions, which means that the resources are strengthened sequentially: in the first variant, a self-efficacy enhancement module precedes a perceived social support enhancement module, whereas in the second variant the order is reversed. Throughout the protocol we refer to these study conditions as experimental ones. Only one module is included in the remaining two conditions, which means that a single resource is targeted; either self-efficacy or perceived social support. These conditions are referred to as active controls. We expect that participation in all conditions will lead to significant changes in primary and secondary outcomes from the baseline to posttest. However, a greater decrease in job stress and job burnout (primary outcomes), depression, and traumatic stress symptoms, and greater increase in work engagement (secondary outcomes) at the posttest will be observed among the participants in the experimental conditions than among those in active control conditions. These differences will be maintained at the 6-month and 12-month follow up.

**Fig. 1:**
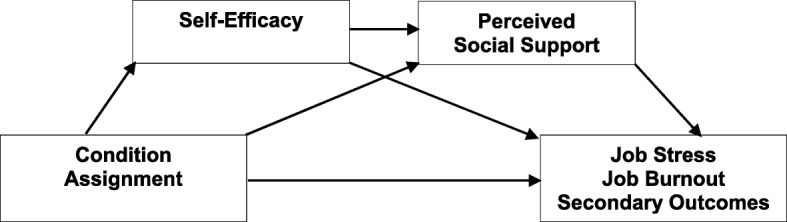
Cultivation hypothesis

**Fig. 2:**
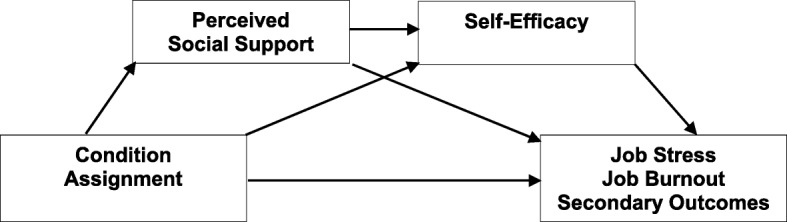
Enabling hypothesis

At the same time, we see no theoretical or empirical grounds to predict which of the two experimental conditions will be more effective than the other in reducing stress and burnout, and thus we consider a comparison between cultivation and enabling effects an explorative part of the study (see Figs. 1 and 2).

Moreover, we make predictions on the mechanisms behind the impact of the study condition assignment on the primary and secondary outcomes. We hypothesize that self-efficacy and perceived social support will mediate - either individually or sequentially - the relationship between condition assignment and the outcomes: the impact of condition assignment on the primary and secondary outcomes posttest will be mediated by (1) self-efficacy or (2) perceived social support, or (3) sequentially by either self-efficacy and perceived social support or perceived social support and self-efficacy. The increase in self-efficacy and perceived social support will lead to a decrease in job stress and job burnout and in depression and secondary traumatic stress symptoms, and to an increase in work engagement posttest.

## Methods

### Design

We will conduct a four-arm randomized controlled trial in parallel design, comparing the effects of (1) self-efficacy and perceived social support sequential enhancement (experimental condition reflecting cultivation hypothesis), (2) perceived social support and self-efficacy sequential enhancement (experimental condition reflecting enabling hypothesis), (3) self-efficacy, and (4) perceived social support enhancement (active controls).

The study has been approved by a research ethics committee and registered at ClinicalTrials.gov on 23 March 2018 (study identifier NCT03475290). The registration process (including obtaining informed consent) and the entire study will be conducted online at medstres.pl.

### Study population

The study is aimed at medical professionals such as physicians, nurses, midwives, physical therapists, and paramedics. Participants will be required to meet the following inclusion criteria: (1) be at least 18 years old and (2) be pursuing an occupation related to health care. Lack of access to a device with an Internet connection will be an exclusion criterion. In line with national surveys, 84.2% of households and 95.6% of private and public entities in Poland have access to the Internet and mobile Internet devices are in common use [42]. However, 11% of medical professionals who participated in our pre-implementation study declared a lack of Internet connection at the workplace. Thus, this criterion has been set as a point for consideration for those potential participants who would be interested in using Med-Stress during working hours only. Participants do not need to meet any criteria for job stress or burnout levels.

### Sample size

The study will be conducted among professionally active health professionals at risk of job stress and burnout. Bonferroni’s adjustment for multiple comparisons will be applied: there will be a comparison between the two experimental conditions and between each experimental condition and each active control condition giving a total of five comparisons posttest. Furthermore, we will include five outcomes. Taken together, this will lower *p* levels to .002. Meta-analyses of the effectiveness of web-based interventions for stress reduction found a small effect of *g* = 0.30 [19] for work-related stress, and a medium size effect of *d* = 0.49 for general stress [43]. Taking into account the minimum effect size of *d* = 0.20, in analysis of covariance (ANCOVA) with a significance level of α = 0.002, statistical power of 0.90, and the number of conditions (two experimental conditions and two active controls), a total of 607 participants will be set (152 participants per condition). Furthermore, a high dropout rate is usually encountered in unguided interventions (between 5 and 45%; [44]) and thus we ultimately plan for a sample of 1200 participants (300 participants per condition).

### Procedure

**Fig. 3:**
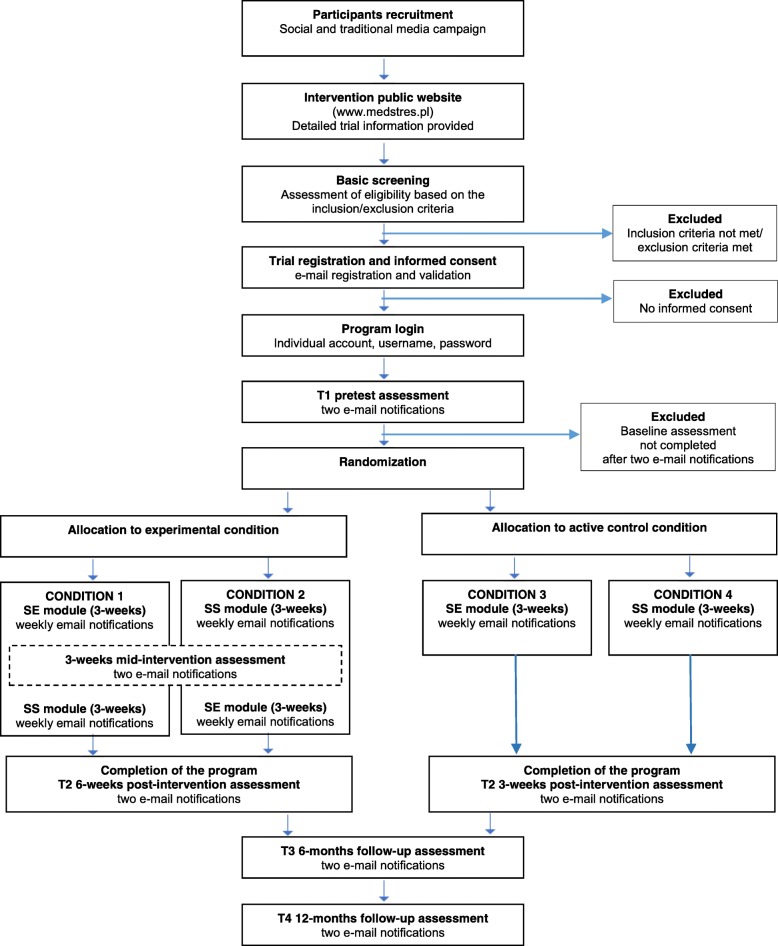
Study flow. SE, Self-efficacy enhancement module; SS, Social support enhancement module

**Fig. 4:**
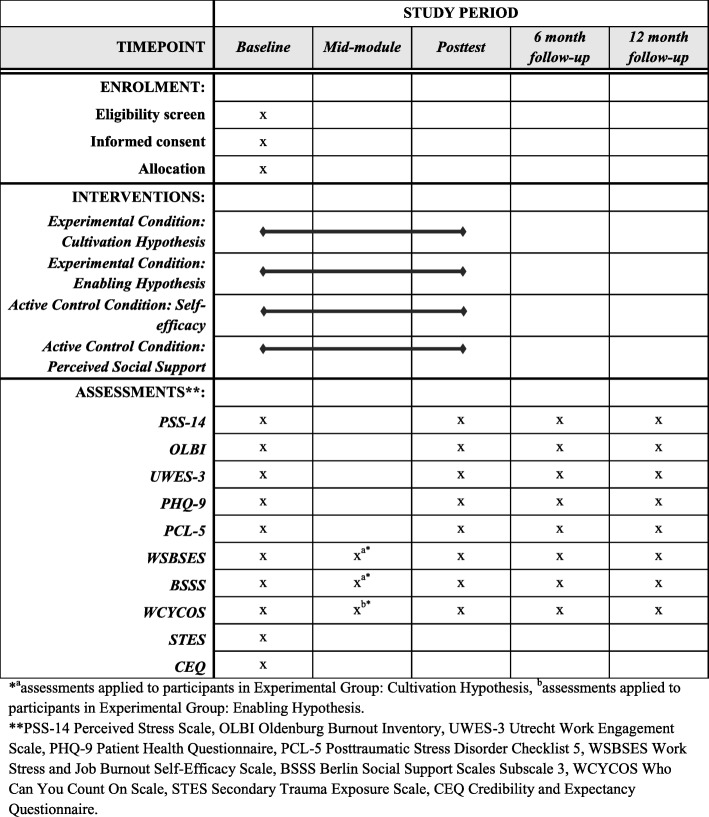
Schedule of enrolment, interventions, and assessments

Study flow is presented in Fig. 3 and the schedule of enrolment, interventions, and assessment (SPIRIT figure) in Fig. 4. The Trial Registration Data checklist and SPIRIT checklist can be found in Additional files 1 and 2, respectively.

Participants will be recruited via targeted Facebook ads, the project’s website and through promotion in the regional and national media. Interested participants will be invited to visit the intervention public page (medstres.pl) and watch a short video clip in which the project’s team explains the purpose and scope of the study. Subsequently, they will be asked to declare whether they are at least 18 years old, represent medical professions, and have access to an Internet-connected device; all in order to check whether they are eligible to participate. Upon registration, participants will be asked to provide informed consent. All registration procedures will be conducted online, with no contact from the research team. The implications of participation in the study will be clearly explained in the materials (text and a video clip) on the public website and in the informed consent document. This process was approved by the research ethics committee. After accepting to be randomized to one of four study conditions, participants will be asked to fill out the baseline questionnaires (pretest). Once those are completed, participants will be randomized and allocated with a 1:1:1:1 ratio to one of four study conditions. The registration process is continuous, which means that access to the Med-Stress program is given instantly, but the content is released on Mondays. Every week a new exercise is available and participants will be notified via e-mail. Such a recurrent design is determined by the task that requires participants in three conditions (those that include a perceived social support enhancement module) to take part in a forum discussion at the same time. Depending on the study condition, the intervention will take 3 weeks (active control conditions) or 6 weeks (experimental conditions). Participants will not be informed about the result of the allocation but it is public knowledge that Med-Stress consists of four variants that differ in terms of length (depending on whether a given variant contains one or two modules) and the type of exercises. Regardless of the condition, all participants have access to four optional modules throughout the study: Relaxation, Mindfulness, Cognitive Restructuring, and Lifestyle. Participants will be asked to fill out questionnaires at four times during the study: before the intervention, posttest (i.e., right after the intervention: at 3 or 6 weeks depending on the study condition), and at 6-month and 12-month follow up. Additionally, users assigned to experimental conditions will undergo mid-intervention assessment. Depending on the first module, perceived social support (one scale) or self-efficacy (two scales: self-efficacy to manage job stress and burnout, and self-efficacy to obtain social support) will be measured. At each assessment point, participants will receive an e-mail notification containing a link to the survey. All measurements throughout the study will be obtained online, which has been shown to be a reliable mode of administering psychological, self-report questionnaires [45]. To ensure a high response rate, each e-mail notification will be followed by two reminders every 2 days. Moreover, we will include supportive messages in the survey between questionnaires, thanking the participants for getting this far and encouraging them to continue until completion. All participants will be advised that should they experience any psychological discomfort, harm, or other unintended effects of the intervention or study, they are encouraged to contact the study team or other mental-health professionals. At any time during the intervention period and after completing the program, the participants have the right to withdraw their participation without the obligation to provide their reasons, as stated in the informed consent.

### Intervention: Med-Stress

Med-Stress is an unguided open access, standalone Internet-based intervention that consists of four conditions: two experimental and two active controls, to which participants will be randomly assigned. In the experimental conditions, participants will complete two obligatory modules (enhancing self-efficacy and perceived social support or perceived social support and self-efficacy), whereas in the active control conditions they will complete only one of them (enhancing self-efficacy or perceived social support). Each module contains three evidence-based, CBT-framed exercises. Therefore, participants in the experimental conditions will be offered six exercises spaced out over 6 weeks, while the participants in the active controls will be presented with three exercises over the span of 3 weeks. Each exercise will be released once a week and users can work on them at their own pace. Participants will be encouraged to do all tasks, but the new exercise will be made available regardless of the completion of the previous one. Each exercise in each module is preceded with a psychoeducation part delivered via a short, animated movie. Participants will not need to watch the movies in order to complete the exercises, they will be only encouraged to do so. Completion of each model - defined as watching the movie, reading the instructions, and providing written responses online - takes about 1.5 h of screen time per week. Some of the exercises (i.e., *vicarious experience and action planning*) require performing actions away from the computer - in their work environment - and one exercise (*social skills and peer support*) necessitates longer screen time to take part in the forum discussion. Additionally, participants will have the option of benefitting from four additional modules at any time during the intervention.

#### Experimental condition: self-efficacy and perceived social support enhancement modules (cultivation hypothesis)

This experimental condition reflects the cultivation hypothesis and comprises two sequential modules with a self-efficacy enhancement module (SE) preceding a perceived social support enhancement module (SS). The SE module includes the following exercises: (1) *mastery experience* in which participants will recall own previous successes at work and identify coping mechanisms that could be used in the future, (2) *vicarious experience* in which participants will be encouraged to find a person at work that they identify with (a model) and recognize that person’s coping mechanisms that could be transplanted into their own life, and (3) *action planning* in which participants will create a plan and identify barriers and ways to overcome them using an “if-then” approach. The SS module includes the following exercises: (1) *received support & cognitive distortions* in which participants will identify situations when help was received, recognize cognitive distortions, and learn about their mechanisms, 2) *social skills & peer support* in which participants will receive and provide support for the other trial participants during a forum discussion in a private (3–4 users) chatroom, and (3) *action planning* in which participants will create a plan and identify barriers and ways to overcome them using an “if-then” approach. This condition lasts 6 weeks: each exercise will be released at the beginning of each week. Moreover, participants will have the option to engage in one or more out of four additional modules: relaxation, mindfulness, cognitive restructuring, and lifestyle.

#### Experimental condition: perceived social support and self-efficacy enhancement modules (enabling hypothesis)

This experimental condition reflects the enabling hypothesis and consists of the same modules as the first experimental condition (cultivation hypothesis) but in reverse order. Participants will first be presented with exercises comprising the SS module, and then the SE module. Participation in this condition also lasts 6 weeks with each exercise being released at the beginning of every week. Similarly, as in the previous condition, participants can optionally engage in one or more out of four additional modules: relaxation, mindfulness, cognitive restructuring, and lifestyle.

#### Active control condition: self-efficacy enhancement module

In this active control condition participants will only be presented with the SE module comprising the same three evidence-based, CBT-framed exercises as in the experimental conditions. Participants will also have access to the same four optional modules. This condition only takes 3 weeks, with one exercise being released each week.

#### Active control condition: social support enhancement module

In the second active control condition participants will only be presented with the SS module comprising the same three evidence-based, CBT-framed exercises as in the experimental conditions. Here again, participants will have access to four optional modules. This condition lasts 3 weeks, with one exercise being released each week.

#### Optional modules

##### Relaxation

This module consists of four voice-guided exercises: (1) breathing, (2) progressive relaxation, (3) visualizing the body’s warmth and weight, and (4) visualizing a calm place. The module aims to provide tools for active relaxation and helps to decrease muscular tension and strain. Participants will be free to do any number of these exercises at any time during the intervention.

##### Mindfulness

This module consists of four voice-guided or text-guided exercises: (1) how do body and mind react to thoughts? (2) body scanning, (3) breathing and sounds, and (4) being mindful of emotions. All exercises are designed to strengthen attention, body, thoughts, and emotional awareness to reduce perceived stress. Again, participants can do any chosen number of these exercises throughout the intervention.

##### Cognitive restructuring

Three exercises constitute this module: (1) opinion or fact, (2) identifying thinking traps, and (3) how important will this be in the future? This module provides an insight into the CBT technique of identifying and altering stress-inducing thoughts. Participants can choose one exercise or do all of them at any point during the intervention.

##### Lifestyle

This module consists of two exercises: (1) physical activity and (2) pleasant activities, which encourage participants to plan and implement activities fostering stress reduction. Users can do only one or both of these exercises throughout the intervention.

### Platform

The Med-Stress intervention was built on the Iterapi Internet platform [46], developed and administered at Linköping University in Sweden. The platform is widely used for international research on psychological Internet-based interventions. It provides professional software, secure transfer (https protocol), and data collection and storage protection required by local and European Union (EU) law, including General Data Protection Regulation (GDPR) (EU/2016/679). All data collected during the intervention and accompanying assessments, including the participants’ consent will be entered directly on the platform by participants and automatically encrypted, coded, and stored on the Iterapi server. Data stored will be depersonalized and masked, with access for authorized persons only.

### Randomization and blinding

Randomization will be carried out using an online randomization program (randomizer.org). Non-stratified block randomization with a 1:1:1:1 allocation ratio will be used to ensure an equal number of participants in all four study conditions (fixed blocks of 4). Participants will not be informed about the condition to which they are assigned, however they will not be blinded to their allocation, as it is publicly known that this intervention consists of four alternatives that differ in duration.

### Outcomes

#### Primary outcomes

There will be two primary outcomes in this study: job stress and job burnout.

The Perceived Stress Scale 14 (PSS-14) [2] will be used to measure job stress. We adapted the questionnaire’s instruction to refer to occupational stress. It consists of 14 items with the response scale ranging from 0 (never) to 4 (very often). A higher total score represents higher perceived stress.

The Oldenburg Burnout Inventory (OLBI) [47] will be applied to measure job burnout. It consists of 16 items with the response scale ranging from 1 (strongly agree) to 4 (strongly disagree). The questionnaire consists of two subscales: exhaustion and disengagement. A higher total score represents greater job burnout.

#### Secondary outcomes

Secondary outcome measures in this study include depression, work engagement and job-related traumatic stress.

The Patient Health Questionnaire (PHQ-9) [48] will be used to measure depression. This scale consists of 9 items and the responses range from 0 (not at all) to 3 (nearly every day). A higher total score represents greater depression.

The Utrecht Work Engagement Scale (UWES-3) [49] will be used to measure work engagement. It is the ultra-short version of the scale, which consists of 3 items and the response scale ranges from 0 (never) to 6 (always). A higher total score represents greater work engagement.

The Posttraumatic Stress Disorder Checklist 5 (PCL-5) [50] will be applied to measure traumatic stress symptoms. The questionnaire consists of four subscales: intrusions, avoidance, negative alterations in cognition and mood, and arousal. The response scale for 20 items range from 0 (not at all) to 4 (extremely). A higher total score represents higher traumatic stress. We adapted the instruction to refer to job-related indirect trauma exposure.

#### Other measures

Additionally, in this study we will measure self-efficacy to manage job burnout and work stress, social support self-efficacy, perceived social support, secondary trauma exposure, and initial expectancy of improvement and perceived credibility of the intervention.

The Work Stress and Job Burnout Self-Efficacy Scale (WSBSES) [51] will be used to measure the first type of self-efficacy. It consists of 28 items with the response scale ranging from 1 (I am definitely not capable) to 7 (I am definitely capable). A higher total score represents greater self-efficacy.

The Berlin Social Support Scales, Subscale 3 (BSSS) [52] will measure social support self-efficacy. The scale was originally designed to measure social support but we adapted its instruction and response scale to reflect self-efficacy for obtaining social support. The scale comprises 5 items and the revised response scale ranges from 1 (I am definitely not capable) to 7 (I am definitely capable). A higher total score represents greater social support self-efficacy.

The Who Can You Count On Scale [53] will measure perceived social support. It consists of 32 items with the response scale ranging from 1 (to very little extent) to 5 (to great extent). The questionnaire consists of 4 subscales: support form supervisors, support from coworkers, support from friends, and support from family. A higher total score represents greater perceived social support.

The Secondary Trauma Exposure Scale (STES) [54] will be used to measure job-related indirect exposure to traumatic events. The scale consists of 13 items with a yes/no response scale for 10 items, 2 items with a response scale ranging from 1 (none/never) to 7 (a few thousands/every day), and one item from 0% to 100%. A higher total score represents greater indirect exposure. Additionally, we will assess direct exposure to traumatic events with a single question on the number of events listed in the STES (response scale from 0 to 10).

The Credibility and Expectancy Questionnaire (CEQ) [55] will be applied to measure users’ expectancy and credibility of the intervention. This scale consists of 6 items, with a response scale for 4 items ranging from 1 (not at all) to 9 (very), and for the remaining 2 items from 0% to 100%. A higher total score represents higher treatment credibility and the user’s expectancy of improvement.

All the scales will be measured pretest, posttest, and at the two follow ups (at 6 and 12 months). The exceptions are STES and CEQ, which will only be assessed at baseline. All scales included have been validated in Poland and their reliability was tested in a feasibility study with satisfying results, with Cronbach’s alpha between 0.75 and 0.97. Completion of the surveys takes about 20 min.

### Statistical analysis

We will conduct analyses of covariance (ANCOVA) with condition assignment as a factor, all primary and secondary measures at posttest as outcomes, and their pretest values as covariates. This will allow us to test for group differences between study conditions posttest while controlling for change over time. ANCOVA has been shown to be more parsimonious than analysis of variance (ANOVA) in randomized trials with a repeated measures design, and thus to have more power [56]. In terms of specific analyses, primarily, we will compare the effects of experimental conditions to each active control to test whether a two-module intervention is more effective that a single-module one. Next, we will compare the effects of the two experimental conditions to check if the sequence of the modules contributes to the intervention effects. Bonferroni’s adjustment for multiple comparisons (five comparisons and five outcomes) will be applied, which will lower the *p* levels to .002 for detection of the effects. Additionally, we will conduct separate ANCOVA to test whether the differences in outcomes between study conditions are maintained at the 6-month and 12-month follow up. Furthermore, we will conduct mediation analyses to verify the hypothesized mechanism of the effect of condition assignment on the primary and secondary outcomes. Sequential mediation analyses will be applied using the PROCESS macro [57] with condition assignment as a predictor, primary and secondary measures posttest as the outcomes, their baseline scores as covariates, and self-efficacy and perceived social support posttest as mediators (see Figs. 1 and 2 for conceptual models of mediations).

Additionally, we will carry out moderation analysis to test whether the initial (pre-intervention) expectancy about the positive outcomes of the treatment (i.e., how successful the intervention would be in reducing stress and burnout symptoms in participants’ opinions) moderates the relationship between the condition assignment and the study outcomes. As participants in the study will have the option of engaging in one or more out of four additional modules, we will consider participation in them as a control variable.

Analyses will be conducted on the intention-to-treat (ITT) sample: all randomized participants will be included in the analyses regardless of their adherence and completion of the intervention. Per-protocol analyses in the sample of those participants who provide responses posttest will additionally be performed. Results of ITT and per-protocol analyses will be compared to check the robustness of the effects. Range and randomness of the missing data will be assessed with Little’s missing completely at random test [58]. Furthermore, we will conduct a dropout analysis comparing the baseline scores of those participants that remained in the study and those that dropped out, using ANOVA for continuous variables and χ^*2*^ tests for categorical variables. Dropout will be defined as those participants who did not fill out measurements beyond pretest. Missing data will be imputed by the means of multiple imputation. Because study outcomes are assessed three times, we will impute missing data using the baseline measurements as predictors, the measurements of the subsequent outcomes and the baseline covariates that could potentially be responsible for the missing data [59].

## Discussion

In this study we will test the efficacy of the four variants of a resource-based intervention, Med-Stress, in reducing job stress and burnout among medical professionals. We expect that participants assigned to the conditions in which two psychological resources, namely self-efficacy and perceived social support or perceived social support and self-efficacy, are enhanced sequentially, will experience a greater decrease in job stress and burnout posttest compared with participants assigned to the conditions strengthening only one of these resources. We expect that these effects will be maintained at 6-month and 12-month follow up.

This study has several strengths. The content of the intervention is theory-driven: it is framed within the conservation of resources theory [20] and allows for the experimental verification of enabling and cultivation effects of self-efficacy and perceived social support. Focus on the psychological resources makes this intervention accessible to a wide array of health professionals, regardless of the specific demands they face at their respective workplaces. In order to facilitate implementation of the intervention and to make it as relevant to the target audience as possible, we conducted a pre-implementation study among medical professionals (*N* = 744). Among other things, we asked about the demands and highlights of their jobs, and subsequently used these answers to design the intervention. For instance, because our responders stated that working with patients is one of the most satisfying parts of their jobs, Med-Stress does not contain any exercises that refer to the “difficult patient” trope. The pre-implementation study also allowed us to build a base of potential users for the program itself. There is no passive control group in our study. Instead we introduce two active controls, each enhancing a single resource. Thus, we aim not at demonstrating that this intervention is more effective than no activity, but rather that targeting two resources (and perhaps in a particular order) is more beneficial in comparison with what we already know works, i.e., strengthening either self-efficacy or perceived social support alone [60]. We introduce two follow up measurement points, at 6 and 12 months, which will allow us to test long-term effects. Research results on Internet-based interventions indicate that they are more effective when an element of the human factor is present even if it constitutes only e-mail reminders [43]. In this study, the exercises in each module will be released weekly and the participants will be informed about it via e-mail. Moreover, invitations to fill out the questionnaires posttest and at follow up are personal and convey the message on why it is important that the surveys are completed. We hope that these interactions, albeit only one-way ones, will increase participants’ engagement. Apart from personalized messages, we introduce other ways in which participants can customize the way they use Med-Stress, and therefore foster their involvement. First, we designed four additional modules that are all optional and can be used at any time during the program. These are Relaxation, Mindfulness, Cognitive Restructuring, and Lifestyle. Second, we made psychoeducation optional and presented it in the attractive form of animated videos. It is possible to do the exercises without the education part, although participants will be informed that they might find watching the videos beneficial. Finally, Med-Stress has been designed specifically for health workers, a group of professionals whose mental health is a matter of public interest.

The study has some known limitations. First, this is an unguided intervention, open to participants that meet the inclusion criteria. Although registration is required, this is essentially an open-access program with the associated pros and cons (for a review, see [61]). The potential problems that we expect are adherence and dropout. Still, we argue that this kind of intervention is appropriate given the target audience and the cultural context. As there is poor institutional support for medical professionals and high stigma surrounding mental health conditions at work, we expect that introducing a low-intensity, highly accessible, free-of-charge program that grants confidentiality will be beneficial to many health workers. Future studies might attempt to cooperate with the management of institutions that hire healthcare providers and deliver to them closed and guided interventions. Second, because Med-Stress is one of the first Internet-based interventions delivered in Poland, we expect that it will probably attract a specific group of people that are relatively highly educated and not intimidated by using the Internet to manage their mental health. Therefore, the sample might be non-representative but at the same time, all medical professionals are required to graduate with a university degree and many of them use computers and the Internet in their daily work.

If successful, this study will contribute to findings on the role of resources in the development of job stress and job burnout by experimentally demonstrating the enabling versus cultivation effects of self-efficacy and perceived social support. We will report on the study results, regardless of what they are, in accordance with the latest version of the Consolidated Standards of Reporting Trials (CONSORT) statement. The most effective of the four tested study conditions could be broadly implemented among medical professionals as a standalone, accessible, and cost-effective intervention for the prevention and management of job stress and burnout.

## Additional files

Additional file 1: **Table S1.** Trial registration data. (DOCX 17 kb)

Additional file 2: SPIRIT 2013 Checklist: Recommended items to address in a clinical trial protocol and related documents. (DOCX 39 kb)

## Abbreviations

ANCOVA: Analysis of covariance
ANOVA: Analysis of variance
BSSS: Berlin Social Support Scales
CBT: Cognitive-behavioral therapy
CEQ: Credibility and Expectancy Questionnaire
GDPR: General Data Protection Regulation
ITT: Intention-to-treat
OLBI: Oldenburg Burnout Inventory
PCL-5: Posttraumatic Stress Disorder Checklist 5
PHQ-9: Patient Health Questionnaire
PSS-14: Perceived Stress Scale 14;
SE: Self-Efficacy Enhancement Module
SS: Social Support Enhancement Module
STES: Secondary Trauma Exposure Scale
UWES-3: Utrecht Work Engagement Scale
WSBSES: Work Stress and Job Burnout Self-Efficacy Scale
