# Supplementary material for: Efficacy of an Internet-based intervention for job stress and burnout among medical professionals: study protocol for a randomized controlled trial
Source: Trials. 2019 Jun 10;20:338. doi: 10.1186/s13063-019-3401-9 (PMC6558742; doi:10.1186/s13063-019-3401-9)
Supplement: Supplementary file 1 — Table S1. Trial registration data. (DOCX 17 kb) [file 13063_2019_3401_MOESM1_ESM.docx]

Table S1. Trial registration data

| Data category | Information |
| --- | --- |
| Primary registry and trial identifying number | ClinicalTrials.gov NCT03475290 |
| Date of registration in primary registry | March 23, 2018 |
| Secondary identifying numbers | I.N.16 Med-Stress |
| Source(s) of monetary or material support | The National Centre for Research and Development, Poland,  Central Institute for Labour Protection - National Research Institute, Poland |
| Primary sponsor | University of Social Sciences and Humanities, Warsaw, Poland |
| Secondary sponsor(s) | N/A |
| Contact for public queries | medstres@swps.edu.pl |
| Contact for scientific queries | PI Ewelina Smoktunowicz, PhD, University of Social Sciences and Humanities, Warsaw, Poland, esmoktunowicz@swps.edu.pl |
| Public title | Internet-Based Intervention for Occupational Stress Among Medical Professionals (Med-Stress) |
| Scientific title | [Project Title: Innovative internet-based, educational and therapeutic program preventing negative consequences of occupational stress among medical professionals.] |
| Countries of recruitment | Poland |
| Health condition(s) or problem(s) studied | Psychological stress, occupational stress, professional burnout |
| Intervention(s) | Behavioral: resources' enhancement: self-efficacy and perceived social support (arm: experimental) |
|  | Behavioral: resources' enhancement: perceived social support and self-efficacy (arm: experimental) |
|  | Behavioral: resources' enhancement: self-efficacy (arm: active control) |
|  | Behavioral: resources' enhancement: perceived social support (arm: active control) |
| Key inclusion and exclusion criteria | Ages eligible for study: ≥18 years; Sexes eligible for study: both; Accepts healthy volunteers: yes |
|  | Inclusion criteria: adults (≥ 18 years), professionally active medical providers |
|  | Exclusion criteria: no access to a device with Internet connection |
| Study type | Interventional |
|  | Allocation: randomized; Intervention model: parallel assignment; Masking: none (open label) |
|  | Primary purpose: prevention |
| Date of first enrollment | October 2018 |
| Target sample size | 1200 |
| Recruitment status | Recruiting |
| Primary outcome(s) | Change on perceived stress (Perceived Stress Scale 14, PSS-14) and job burnout (Oldenburg Burnout Inventory, OLBI); Time Frame: baseline, 3 or 6 weeks depending on the study condition, 6 months, 12 months |
| Key secondary outcomes | Change on work engagement (Utrecht Work Engagement Scale, UWES-3), depression (Patient Health Questionnaire, PHQ-9), and posttraumatic stress symptoms (Posttraumatic Stress Disorder Checklist 5, PCL-5); Time Frame: baseline, 3 or 6 weeks depending on the study condition, 6 months, 12 months |
| Ethics review | Approved by a human research ethics committee: the Institutional Review Board at the SWPS University of Social Sciences and Humanities: No 4/2018 13^th^ February 2018. |
